# Reversal of intrinsic multidrug resistance in Chinese hamster ovary cells by amiloride analogs.

**DOI:** 10.1038/bjc.1991.58

**Published:** 1991-02

**Authors:** R. F. Epand, R. M. Epand, R. S. Gupta, E. J. Cragoe

**Affiliations:** Department of Biochemistry, McMaster University, Hamilton, Ontario, Canada.

## Abstract

A number of amiloride analogs can sensitise wild type Chinese Hamster ovary (CHO) cells to the cytotoxic action of vinblastine, daunomycin, puromycin or colchicine. Some of these analogs also have weak sensitising effects on the multidrug resistant CHO cell line, CHRC5. The unusual feature of most of the active amiloride analogs is that they are more potent in reversing the intrinsic multidrug resistance (MDR) phenotype of CHO cells than their acquired MDR characteristic. Human HeLa cells that do not exhibit intrinsic MDR are not affected by these agents. Several of the amiloride analogs have a greater effect in increasing adriamycin uptake in wild type CHO cells than they do with CHRC5 cells. The differential effect of amiloride analogs on intrinsic versus acquired MDR characteristics of Chinese hamster cells suggests some differences in the underlying resistance mechanisms.


Br. J. Cancer (1991), 63, 247-251 © Macmillan Press Ltd., 1991

### Reversal of intrinsic multidrug resistance in Chinese hamster ovary cells by amiloride analogs

R.F. Epand, R.M. Epand, R.S. Gupta & E.J. Cragoe, Jr*

*Department of Biochemistry, McMaster University, Health Sciences Centre, 1200 Main Street West, Hamilton, Ontario, Canada L8N 3Z5.*

**Summary** A number of amiloride analogs can sensitise wild type Chinese Hamster ovary (CHO) cells to the cytotoxic action of vinblastine, daunomycin, puromycin or colchicine. Some of these analogs also have weak sensitising effects on the multidrug resistant CHO cell line, $CH^RC5$. The unusual feature of most of the active amiloride analogs is that they are more potent in reversing the intrinsic multidrug resistance (MDR) phenotype of CHO cells than their acquired MDR characteristic. Human HeLa cells that do not exhibit intrinsic MDR are not affected by these agents. Several of the amiloride analogs have a greater effect in increasing adriamycin uptake in wild type CHO cells than they do with $CH^RC5$ cells. The differential effect of amiloride analogs on intrinsic versus acquired MDR characteristics of Chinese hamster cells suggests some differences in the underlying resistance mechanisms.

Cells can be selected for resistance to cytotoxic drugs. Frequently this drug resistance will be pleiotropic. One class of mutant selected in mammalian cells exhibits increased resistance to a variety of naturally occurring and semi-synthetic cytotoxic drugs including vinca alkaloids, colchicine, adriamycin, daunomycin and puromycin (Beidler & Meyers, 1989; Endicott & Ling, 1989; Juranka *et al.*, 1989). This form of multidrug resistance (MDR) has been referred to as acquired resistance. In addition, it has recently been shown that certain wild type cells, particular those derived from rodents, can be made more sensitive to cytotoxic drugs by agents (e.g. verapamil) which reverse MDR (Gupta, 1988). This expression of resistance, to the same group of drugs as seen in the MDR mutant, does not require prior selection with cytotoxic agents and has been termed intrinsic MDR (Gupta, 1988). In this work, for the first time, we show that several analogs of amiloride can reverse intrinsic MDR without greatly affecting the acquired MDR.

Amiloride is an inhibitor of $Na^+/H^+$ antiport. The activity of this ion transporter is elevated in drug resistant cell lines (Boscoboinik *et al.*, 1990). Analogs of amiloride vary greatly in the potency and specificity by which they inhibit this antiporter (Kleyman & Cragoe, 1988). Inhibition of $Na^+/H^+$ transport leads to acidification of the intracellular pH ($pH_i$). Positively charged cytotoxic drugs which can permeate the cell membrane will accumulate on the acidic side of the membrane. Since $pH_i$ is elevated in resistant cells compared to wild type cells (Keizer & Joenje, 1989; Boscoboinik *et al.*, 1990), it seemed possible that acidification of $pH_i$, through inhibition of the $Na^+/H^+$ antiporter, could contribute to the sensitisation of drug resistant cell lines.

### Materials

Amiloride analogs were synthesised for this study by previously described methods (Cragoe *et al.*, 1967). Minimum essential medium (α-MEM) containing L-glutamine and all four ribonucleosides and α-deoxyribonucleosides (α-MEM + nucleosides), faetal bovine serum, penicillin, streptomycin, and amphotericin were obtained from Gibco, Grand Island, NY; trypsin from Difco, Detroit, MI; methylene blue from Fisher Scientific Co., Fairlawn, NY; adriamycin, HCl, and vinblastine sulfate were purchased from Sigma Chemical Co., St. Louis, MO.

*Cell lines and culture conditions*

The parental Chinese hamster ovary cell line which requires proline for growth ($Pro^-$) is referred to as wild type (WT) in our work. The cell line AUXB1 which in addition to proline requires glycine, adenosine and thymidine for growth, was derived from $Pro^-$ WT cells by a single mutational alteration (McBurney & Whitmore, 1974). The $Pro^-$ and AUXB1 cell lines show similar sensitivity towards various drugs used in the present study (Gupta, R.S., unpublished results). The cell line $CH^RC5$ was derived from AUXB1 after three successive selections in presence of increasing concentrations of colchicine (Ling & Thompson, 1974). The $CH^RC5$ cell line (Bech-Hansen *et al.*, 1976) was kindly provided by Dr Victor Ling of the Ontario Cancer Institute, Toronto, Ontario. HeLa (clone $S_3$) is a human cell line established from a cervical carcinoma (Puck *et al.*, 1956). All of the above cell lines were grown as monolayer cultures in α-MEM + nucleosides supplemented with 5-10% foetal bovine serum at 37°C in a humidified incubator in an atmosphere of 95% air and 5% $CO_2$. The cell lines were routinely grown in the absence of any selective drug without loss of resistance.

*Clonogenic assay*

The effect of various agents on the reversal of the drug-resistance was examined by determining the cloning efficiencies of the parental and resistant cell lines in the presence of different concentrations of either vinblastine, daunomycin, puromycin or colchicine, in the absence and presence of the amiloride analogs. In these experiments, which were carried out in 24-well tissue culture dishes, 0.5 ml of 11 progressive dilutions of cytotoxic drug (made at two times the final concentrations in growth medium) were added to duplicate wells of 24-well dishes. These dilutions were chosen to cover a range of concentrations both above and below the cytotoxic level either in the presence or in the absence of sensitiser. Generally, 11 different concentrations of the drug, in addition to a control without any drug, were employed. The single cell suspensions of the cell lines were suitably diluted (based on cell count measurement done by Coulter counter), and 0.5 ml of these suspensions, containing 200 or 500 cells together with a fixed concentration of amiloride analog were then added to the wells of 24-well dishes. The experiments were carried out in parallel with and without the reversing agents. Amiloride analog solutions were made in dimethyl sulfoxide (DMSO); vinblastine sulfate solutions were made in ethanol and diluted in medium. The final concentration of solvent present in the wells did not exceed 2% and in most cases was below 0.2%. The control dishes (i.e. without reversing agent) received an equivalent

*Private Consultant, PO Box 1548, Nacogdoches, TX, 75963-1548, USA.
Correspondence: R.M. Epand.
Received 15 May 1990; and in revised form 14 August 1990.

**Table I** Structure and ion transport inhibitory activity of amiloride and its analogs (active compounds indicated by asterisk)

| R | Abbreviation | $K_i$ (μM) for inhibition of ion transport: $Na^+$ Channel | $Na^+/H^+$ | $Na^+/Ca^{2+}$ |
|---|---|---|---|---|
| $NH_2$ | Amiloride | 0.34 | 83.8 | 1100 |
|  | MACMA | >400 | 1.4 | 1571* |
|  | MTBA | – | – | –* |
|  | MIBA | >300 | 0.44 | 100* |
|  | HMA | >400 | 0.16 | 100 |

| R | Abbreviation | $Na^+$ Channel | $Na^+/H^+$ | $Na^+/Ca^{2+}$ |
|---|---|---|---|---|
|  | DCB | 0.10 | >400 | 30 |
|  | Benzamil | 0.06 | >1000 | 100 |
|  | Phenamil | 0.003 | >500 | 100* |
|  | α-Naphthamil | – | – | –* |
|  | Fluorenamil | 0.085 | >500 | 35 |
|  | Dibenzamil | 0.22 | >100 | 10* |

*Continued overleaf*

| R | Abbreviation | $K_i$ (μM) for inhibition of ion transport<br>$Na^+$ Channel | $Na^+/H^+$ | $Na^+/Ca^{2+}$ |
|---|---|---|---|---|
|  | BTMB | – | – | 17.7* |
|  | HPA | 0.019 | >300 | 1100* |
|  | DMP | 0.015 | >300 | 151* |
|  | | | | |
| R1  | CBDMB | >400 | >500 | 7.3* |
| R2  | | | | |
| *Other structure* | *Abbreviation* | Na+<br>*Channel* | *Na+/H+* | *Na+/Ca2+* |
|  | *DBC* | – | – | – * |

amount of the appropriately diluted solvent. At these concentrations, the solvents had no significant effect on cell viability or the number of clones formed. The cytotoxicity of amiloride analogs towards various cell lines was determined in separate clonogenic assays. Only concentrations of amiloride analogs below the 50% cytotoxic level were used in the drug sensitivity tests. The dishes were incubated for 6–10 days (about 6–7 days for CHO and 8–10 days for HeLa cells) at 37°C in 5% $CO_2$–95% air incubator. By this time, each colony in the control wells generally has 100 or more cells. Subsequently, the dishes were stained for about 30 min with 0.5% methylene blue in 50% methanol and the number of colonies (aggregates of >25 cells) in each well was scored. From the average numbers of colonies observed in the presence of different drug concentrations, the $D_{10}$ values (i.e. drug concentrations which reduced cloning efficiency to approximately 10% of that in the absence of any drug), of different cell lines in the absence and presence of various reversing agents were determined. The degree of resistance of any cell line was determined from the ratio of $D_{10}$ values for the mutant *vs* parental cell lines. The sensitising effect of reversing agents was calculated from the ratios of $D_{10}$ values observed in the absence and presence of reversing drug. Each clonogenic assay was repeated in at least two independent experiments. Reproducibility of the $D_{10}$ values in independent assays was generally within 25%.

### *Drug accumulation*

Cellular uptake of adriamycin was measured by fluorescence according to the method of Ganapathi and Grabowski (1983) as modified by Chambers *et al.* (1989). Briefly, cells were grown in culture medium without the addition of drugs until the 10 cm diameter culture dishes were covered with a monolayer of cells (about $9 \times 10^6$ cells). Then a solution of adriamycin in $H_2O$ was added to all plates so as to give a final concentration of 10 μM. Half of the dishes were used as control. The other half, a small volume of an amiloride analog solution in DMSO was added. The solvent concentration was always below 1%. All dishes were incubated at 37°C in a 5% $CO_2$ atmosphere for 0, 1, 2 and 3 h, in duplicate. After the designated periods of incubation, the dishes were washed twice with phosphate buffered saline and then extracted with 9 ml 0.3 M HCl in 50% methanol for 1–2 min. The fluorescence was then measured using the ratio mode in a Perkin Elmer MPF-44 fluorimeter with an excitation of 470 nm and an emission of 585 nm.

### Results

A number of analogs of amiloride were tested for their ability to reverse multidrug resistance in Chinese hamster ovary (CHO) cells. These amiloride analogs (Table I) have been previously characterised for their effects on ion transport (Kleyman & Cragoe, 1988; Cragoe and coworkers, unpublished results). Of the many compounds tested, several had activity as sensitisers (Table II). That is, concentrations of amiloride analogs which alone had minimal effect on the number of colonies formed, sensitised cells to the cytotoxic action of vinblastine. The number of colonies formed in the presence of cytotoxic drug plus amiloride analog was always compared with control wells which contained only the amiloride analog. In general, these compounds were more effective in reversing the intrinsic MDR characteristic of CHO cells than in reversing acquired resistance. Several of the compounds which specifically reversed intrinsic resistance to vinblastine, were tested against other cytotoxic agents (Table III). As with vinblastine, intrinsic but not acquired resistance to these other cytotoxic drugs is reversed by the amiloride analogs. In addition, these sensitisers have no effect on HeLa cells (Table III), a cell line which does not exhibit intrinsic MDR (Gupta, 1988). This was also found when vinblastine was used as the cytotoxic agent (data not shown).

One of the causes for resistance to cytotoxic drugs is a decreased accumulation of cytotoxic agent. This could explain why wild type cells take up more adriamycin than do resistant cells (Figure 1). Panel a shows an enhancement of drug uptake in both WT and $CH^RC5$ cell lines in the presence of DMP or naphthamil. This is consistent with the partial reversal of both intrinsic and acquired resistance by these sensitisers (Table II). However, the effects of phenamil, HPA and MIBA on adriamycin uptake (Figure 1, panel b) are specific for the WT cell line, with no increase in uptake seen with the resistant cell line. These three drugs were effective in reversing only intrinsic and not acquired resistance (Table II).

**Table II** Analogs active in sensitising Chinese hamster ovary cells to vinblastine

| Analog | $D_{50}$[a] (μM) | Concentration μM | Fold sensitisation WT | $CH^RC5$ |
|---|---|---|---|---|
| No addition | | – | 1[b] | 1[b] |
| MACMA | >400 | 100 | 1.4 | 1.1 |
| | | 200 | 3.3 | 1 |
| MTBA | 12 | 10 | 2.0 | 1 |
| MIBA | 12 | 10 | 4.5 | 1 |
| CBDMB | 0.6 | 0.5 | 3 | 1 |
| Phenamil | 50 | 25 | 3.5 | 1 |
| DBC | >50 | 50 | 3 | 4.5 |
| Naphthamil | 6 | 2.8 | 1 | 2.5 |
| | | 5.6 | 2.5 | 10 |
| DMP | 30 | 15 | 5 | 10 |
| BTMB | 5 | 4 | 5 | 2 |
| HPA | 80 | 77 | 10 | 2 |
| Dibenzamil | 3 | 1.8 | 2.5 | 1 |

[a]$D_{50}$ – concentration of amiloride analog which reduces cloning efficiency to 50% of control. [b]Effect of sensitisers compared with same cell line in the absence of drug. The $D_{10}$ (i.e. the vinblastine concentration which reduced the cloning efficiency to approximately 10% of that in the absence of any drug) was 12 nM for WT and 123 nM for $CH^RC5$.

### Discussion

The observation that some of the amiloride analogs are more effective against intrinsic MDR than acquired MDR is of particular interest. These are the only sensitisers that have been shown to affect intrinsic resistance more than acquired resistance. In contrast to these compounds, other agents such as verapamil, reserpine or cyclosporin concomitantly reverse both intrinsic as well as acquired MDR (Tsuruo *et al.*, 1981; Twentyman *et al.*, 1987; Gupta, 1988 and unpublished results). Not only are sensitisers generally common to both intrinsic and acquired resistance, but also the drugs to which the cells are resistant are the same.

The mechanism of acquired MDR has been studied more extensively. Cell lines with acquired MDR overexpress a 170 kDa membrane glycoprotein, the P-glycoprotein (van der Bliek *et al.*, 1986; Fojo *et al.*, 1987; Scheper *et al.*, 1988; Juranka *et al.*, 1989). This P-glycoprotein is believed to function as an ATP-dependent efflux pump for cytotoxic drugs (Juranka *et al.*, 1989). However, it is not clear that this is the sole mechanism for acquired MDR and several discrepancies between the level of P-glycoprotein expression and the level of acquired resistance have appeared in the literature. In addition, little is known about the mechanism of sensitisation or reversal of acquired resistance. It has been suggested that sensitisers increase drug accumulation by competing with cytotoxic drugs for sites on the P-glycoprotein efflux pump (Horio *et al.*, 1988). However, one sensitiser, cyclosporin A, has been shown to reverse acquired MDR in some cell lines but not in DC3F/ADX cells, despite the fact that these cells overexpress P-glycoprotein (Boscoboinik *et al.*, 1990).

**Table III** Sensitisation of Chinese hamster ovary cells to other cytotoxic agents

| Cytotoxic drug | Reversing[a] agent | $D_{10}$ value (μg $ml^{-1}$) and fold sensitisation WT (CHO) | $CH^RC5$ | HeLa |
|---|---|---|---|---|
| Daunomycin | NONE | 0.025 (1) | 0.30 (1) | 0.003 (1) |
| | MTBA | 0.015 (1.7) | 0.30 (1) | 0.003 (1) |
| | MIBA | 0.015 (1.7) | 0.30 (1) | 0.003 (1) |
| | CBDMB | 0.007 (3.6) | 0.30 (1) | 0.003 (1) |
| Puromycin | NONE | 3.5 (1) | 25 (1) | 0.15 (1) |
| | MIBA | 1.4 (2.5) | 25 (1) | 0.15 (1) |
| | CBDMB | 1.1 (3.2) | 25 (1) | 0.15 (1) |
| Colchicine | NONE | 0.045 (1) | 2.0 (1) | 0.002 (1) |
| | CBDMB | 0.017 (2.6) | 2.0 (1) | 0.002 (1) |

[a]MTBA, MIBA and CBDMB were used at the same concentration as indicated in Table II.

**Figure 1** Effect of amiloride analogs on the accumulation of adriamycin by wild type CHO cells (solid lines, open symbols) and drug resistant $CH^RC5$ cells (dashed line, filled symbols). **a**, No drug (●,○); naphthamil (■,□) and DMP (▲,△). **b**, No drug (●,○); MIBA (■,□); HPA (◆,◇) and phenamil (▲,△). Adriamycin concentration: 10 μM; concentration of sensitiser: 10 μM, except for HPA where 77 μM was used.

The mechanism of intrinsic MDR is even less well understood. Based on very similar characteristics of intrinsic MDR and acquired MDR (with regard to drug cross resistance pattern and reversal by agents such as verapamil, reserpine, etc.), the mechanisms responsible for the two should be related. Therefore, differences in P-glycoprotein expression between human and rodent cells may be responsible for the intrinsic MDR phenotype of the latter cells. In this context, our observation that the intrinsic and acquired MDR phenotypes differ with regard to their reversal by amiloride analogs points to some subtle differences in the resistance mechanism. It should however, be mentioned that acquired MDR cell lines which overexpress P-glycoprotein are also known to differ in their reversal characteristics by cyclosporin A (Boscoboinik *et al.*, 1990). The different reversing agents may provide a valuable probe for examining the heterogeneity of the MDR phenotype and for investigating the underlying mechanism(s).

Although some of the drug sensitising amiloride analogs are potent inhibitors of $Na^+/H^+$ antiport, this does not seem to be their mechanism of action since some antiport inhibitors are not sensitisers and some sensitisers are not inhibitors. In addition, although three of the amiloride analogs which act as sensitisers are potent $Na^+$ channel inhibitors, i.e. phenamil, HPA and DMP, this also does not account for the mechanism of action of all of the amiloride analogs. It is, of course, possible that different sensitisers function by different mechanisms. However, a simpler and more likely explanation is that this group of related compounds are among the several hydrophobic and cationic amphiphiles that reverse multidrug resistance and that their action is independent of inhibition of ion fluxes.

We wish to thank Mrs Rajni Gupta for her assistance in performing many of the drug sensitivity tests described in the present work. This work was supported by a grant from the National Cancer Institute of Canada.

### References

BECH-HANSEN, N.T., TILL, J.E. & LING, V. (1976). Pleiotropic phenotype of colchicine-resistant CHO cells: cross-resistance and collateral sensitivity. *J. Cell Physiol.*, **88**, 23.

BIEDLER, J.L. & MEYERS, M.B. (1989). Multidrug resistance (vinca alkaloids, actinomycin D and anthracycline antibiotics). In *Drug Resistance in Mammalian Cells*, Vol. II Gupta, R.S. (ed.), CRC Press: Florida, pp. 57–88.

BOSCOBOINIK, D., GUPTA, R.S. & EPAND, R.M. (1990). Investigation of the relationship between altered intracellular pH and multidrug resistance in mammalian cells. *Br. J. Cancer*, **61**, 568.

CHAMBERS, S.K., HAIT, W.N., KACINSKI, B.M., KEYES, S.R. & HANDSCHUMACHER, R.E. (1989). Enhancement of anthracycline growth inhibition in parent and multidrug-resistant Chinese hamster ovary cells by cyclosporin A and its analogues. *Cancer Res.*, **49**, 6275.

CRAGOE, E.J. Jr, WOLTERSDORF, O.W. Jr, BICKING, J.B., KWONG, S.F. & JONES, J.H. (1967). Pyrazine Diuretics. II. N-Amidino-3-amino-5-substituted-6-halopyrazinecarboxamides. *J. Med. Chem.*, **10**, 66.

ENDICOTT, J.A. & LING, V. (1989). The biochemistry of P-glycoprotein-mediated multidrug resistance. *Ann. Rev. Biochem.*, **58**, 137.

FOJO, A.T., UEDA, K., SLAMON, D.J., POPLACK, D.G., GOTTESMAN, M.M. & PASTAN, I. (1987). Expression of a multidrug-resistance gene in human tumors and tissues. *Proc. Natl Acad. Sci USA*, **84**, 265.

GANAPATHI, R. & GRABOWSKI, D. (1983). Enhancement of sensitivity to adriamycin in resistant P388 leukemia by the calmodulin hibitory trifluoperazine. *Cancer Res.*, **43**, 3696.

GUPTA, R.S. (1988). Intrinsic multidrug resistant phenotype of Chinese hamster (rodent) cells in comparison to human cells. *Biochem. Biophys. Res. Commun.*, **153**, 598.

HORIO, M., GOTTESMAN, M.M. & PASTAN, I. (1988). ATP-dependent transport of vinblastine in vesicles from human multidrug-resistant cells. *Proc. Natl Acad. Sci. USA*, **85**, 3580.

JURANKA, P.F., ZASTAWNY, R.L. & LING, V. (1989). P-glycoprotein: multidrug-resistance and a superfamily of membrane-associated transport proteins. *FASEB J.*, **3**, 2583.

KEIZER, H.G. & JOENJE, H. (1989). Increased cytosolic pH in multidrug-resistance human lung tumor cells: effect of verapamil. *J. Natl Cancer Inst.*, **81**, 706.

KLEYMAN, T.R. & CRAGOE, E.J. Jr (1988). Amiloride and its analogs as tools in the study of ion transport. *J. Membrane Biol.*, **105**, 1.

LING, V. & THOMPSON, L.H. (1974). Reduced permeability in CHO cells as a mechanism of resistance to colchicine. *J. Cell. Physiol.*, **83**, 103.

MCBURNEY, M.W. & WHITMORE, G.F. (1974). Isolation and biochemical characterization of folate deficient mutants of Chinese hamster cells. *Cell*, **2**, 173.

PUCK, T.T., MARCUS, P.I. & CIECIURA, S.J. (1956). Clonal growth of mammalian cells *in vitro*: growth characteristics of colonies from single HeLa cells with and without feeder layers. *J. Exp. Med.*, **103**, 273.

SCHEPER, R.J., BULTE, J.W.M., BRAKKEE, J.G.P. & 8 others (1988). Monoclonal antibody JSB-1 detects a highly conserved epitope on the P-glycoprotein associated with multidrug-resistance. *Int. J. Cancer*, **42**, 389.

TSURUO, T., IIDA, H., TSUKAGOSHI, S. & SAKURAI, Y. (1981). Overcoming of vincristine resistance in P388 leukemia *in vivo* and *in vitro* through enhanced cytotoxicity of vincristine and vinblastine by verapamil. *Cancer Res.*, **41**, 1967.

TWENTYMAN, P.R., FOX, N.E. & WHITE, D.J.G. (1987). Cyclosporine A and its analogs as modifiers of adriamycin and vincristine resistance in a multi-drug resistant human lung cancer cell line. *Br. J. Cancer*, **56**, 55.

VAN DER BLIEK, A., VAN DER VELDE-KOERTS, T., LING, V. & BORST, P. (1986). Overexpression and amplification of five genes in a multidrug-resistant Chinese hamster ovary cell line. *Molec. & Cellular Biol.*, **6**, 1671.


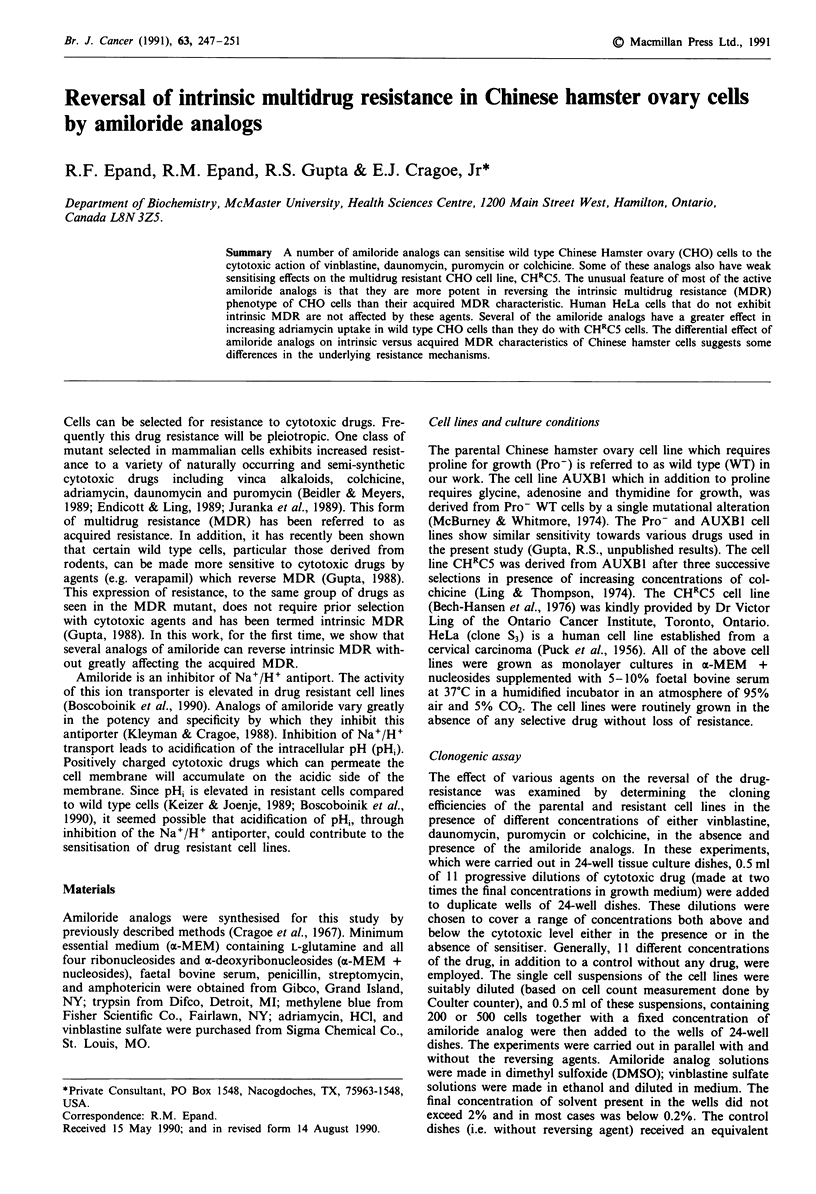

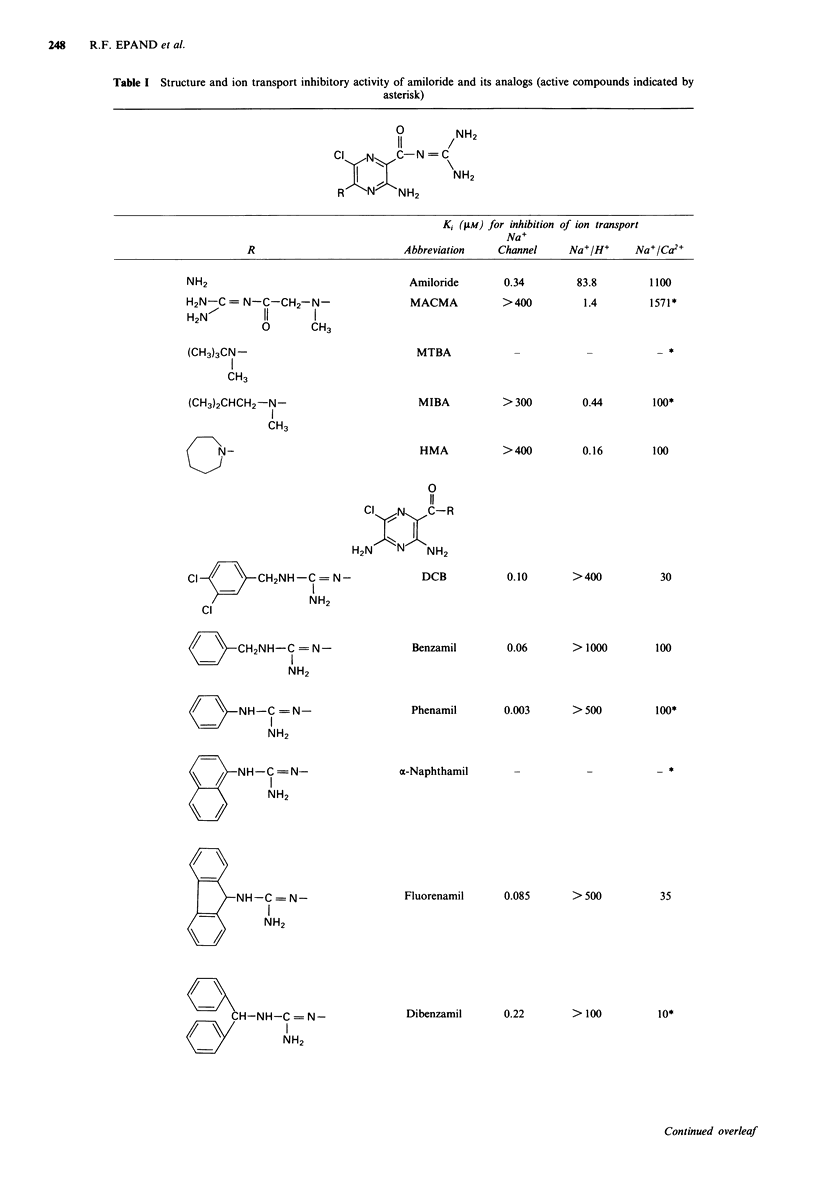

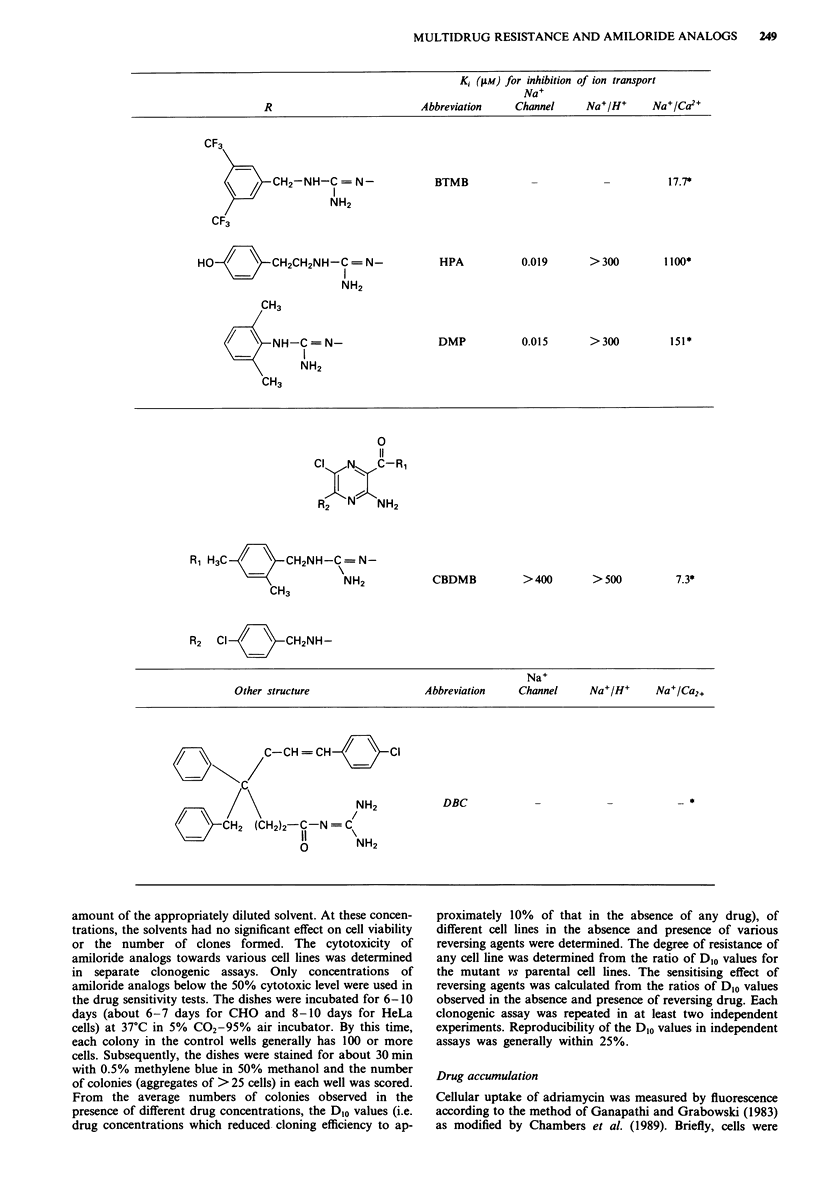

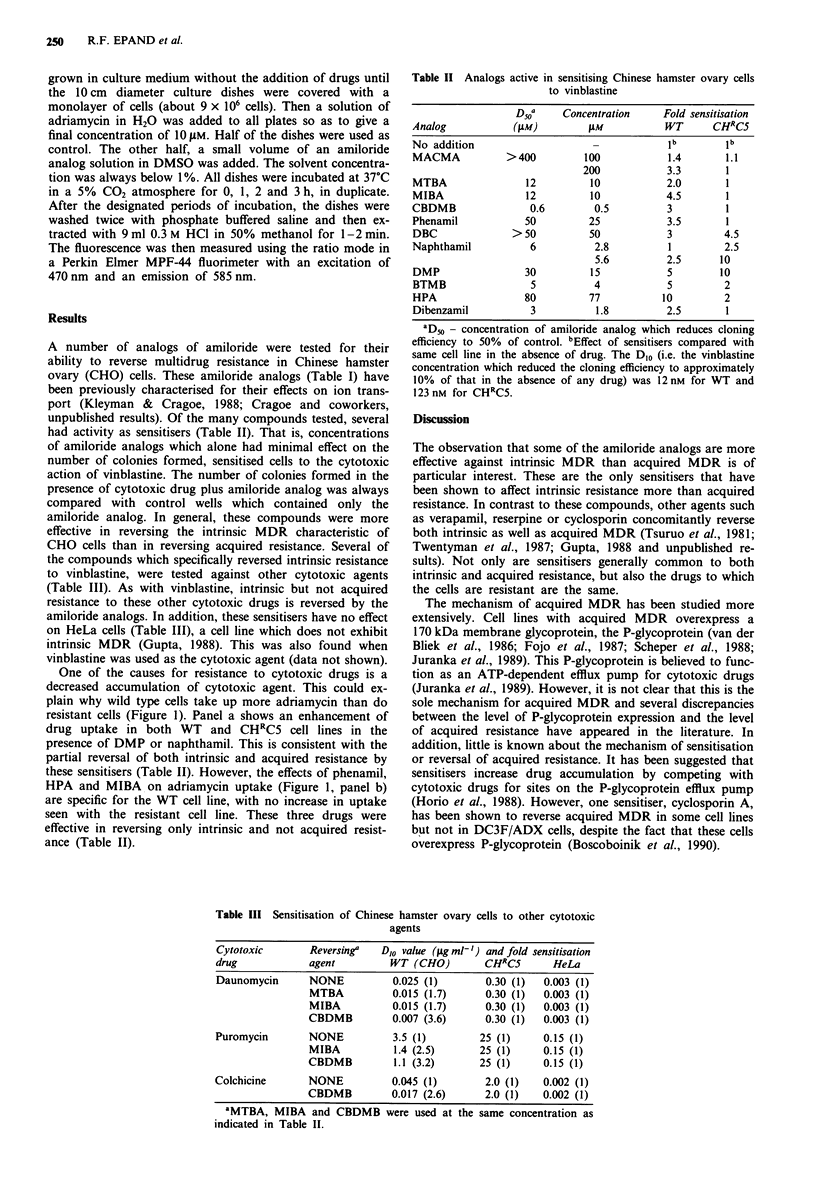

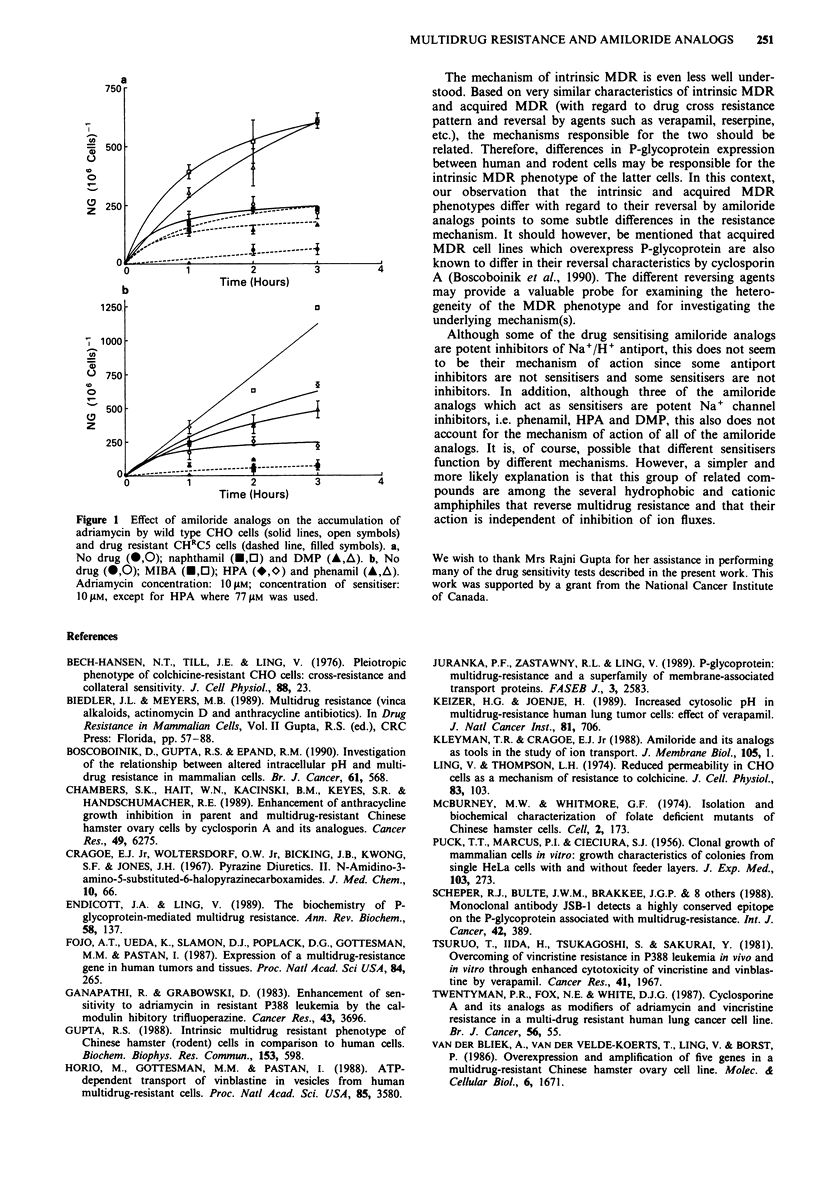


## References

[OCR_00565] Bech-Hansen N. T., Till J. E., Ling V. (1976). Pleiotropic phenotype of colchicine-resistant CHO cells: cross-resistance and collateral sensitivity.. J Cell Physiol.

[OCR_00576] Boscoboinik D., Gupta R. S., Epand R. M. (1990). Investigation of the relationship between altered intracellular pH and multidrug resistance in mammalian cells.. Br J Cancer.

[OCR_00581] Chambers S. K., Hait W. N., Kacinski B. M., Keyes S. R., Handschumacher R. E. (1989). Enhancement of anthracycline growth inhibition in parent and multidrug-resistant Chinese hamster ovary cells by cyclosporin A and its analogues.. Cancer Res.

[OCR_00588] Cragoe E. J., Woltersdorf O. W., Bicking J. B., Kwong S. F., Jones J. H. (1967). Pyrazine diuretics. II. N-amidino-3-amino-5-substituted 6-halopyrazinecarboxamides.. J Med Chem.

[OCR_00594] Endicott J. A., Ling V. (1989). The biochemistry of P-glycoprotein-mediated multidrug resistance.. Annu Rev Biochem.

[OCR_00599] Fojo A. T., Ueda K., Slamon D. J., Poplack D. G., Gottesman M. M., Pastan I. (1987). Expression of a multidrug-resistance gene in human tumors and tissues.. Proc Natl Acad Sci U S A.

[OCR_00605] Ganapathi R., Grabowski D. (1983). Enhancement of sensitivity to adriamycin in resistant P388 leukemia by the calmodulin inhibitor trifluoperazine.. Cancer Res.

[OCR_00610] Gupta R. S. (1988). Intrinsic multidrug resistance phenotype of Chinese hamster (rodent) cells in comparison to human cells.. Biochem Biophys Res Commun.

[OCR_00615] Horio M., Gottesman M. M., Pastan I. (1988). ATP-dependent transport of vinblastine in vesicles from human multidrug-resistant cells.. Proc Natl Acad Sci U S A.

[OCR_00620] Juranka P. F., Zastawny R. L., Ling V. (1989). P-glycoprotein: multidrug-resistance and a superfamily of membrane-associated transport proteins.. FASEB J.

[OCR_00625] Keizer H. G., Joenje H. (1989). Increased cytosolic pH in multidrug-resistant human lung tumor cells: effect of verapamil.. J Natl Cancer Inst.

[OCR_00630] Kleyman T. R., Cragoe E. J. (1988). Amiloride and its analogs as tools in the study of ion transport.. J Membr Biol.

[OCR_00633] Ling V., Thompson L. H. (1974). Reduced permeability in CHO cells as a mechanism of resistance to colchicine.. J Cell Physiol.

[OCR_00638] McBurney M. W., Whitmore G. F. (1974). Isolation and biochemical characterization of folate deficient mutants of Chinese hamster cells.. Cell.

[OCR_00643] PUCK T. T., MARCUS P. I., CIECIURA S. J. (1956). Clonal growth of mammalian cells in vitro; growth characteristics of colonies from single HeLa cells with and without a feeder layer.. J Exp Med.

[OCR_00649] Scheper R. J., Bulte J. W., Brakkee J. G., Quak J. J., van der Schoot E., Balm A. J., Meijer C. J., Broxterman H. J., Kuiper C. M., Lankelma J. (1988). Monoclonal antibody JSB-1 detects a highly conserved epitope on the P-glycoprotein associated with multi-drug-resistance.. Int J Cancer.

[OCR_00655] Tsuruo T., Iida H., Tsukagoshi S., Sakurai Y. (1981). Overcoming of vincristine resistance in P388 leukemia in vivo and in vitro through enhanced cytotoxicity of vincristine and vinblastine by verapamil.. Cancer Res.

[OCR_00661] Twentyman P. R., Fox N. E., White D. J. (1987). Cyclosporin A and its analogues as modifiers of adriamycin and vincristine resistance in a multi-drug resistant human lung cancer cell line.. Br J Cancer.

[OCR_00667] Van der Bliek A. M., Van der Velde-Koerts T., Ling V., Borst P. (1986). Overexpression and amplification of five genes in a multidrug-resistant Chinese hamster ovary cell line.. Mol Cell Biol.

